# Image quality assessments according to the angle of tilt of a flex tilt coil supporting device: An ACR phantom study

**DOI:** 10.1002/acm2.13218

**Published:** 2021-05-02

**Authors:** Ho Beom Lee, Ji Sung Jang, Ki Baek Lee, Sung Min Kim

**Affiliations:** ^1^ Department of Medical Device Industry Dongguk University Seoul South Korea; ^2^ Departments of Radiology and Research Institute of Radiology Asan Medical Center University of Ulsan College of Medicine Seoul South Korea; ^3^ Biomedical Engineering Research Center Asan Institute for Life Sciences Asan Medical Center Seoul South Korea

**Keywords:** flex tilt coil, image quality, MR‐Imaging

## Abstract

In this study, we assessed how image quality depends on the angle of tilt of a flex tilt coil supporting device during an MRI examination. All measurements were performed with an American College of Radiology (ACR) MRI phantom using a flex tilt coil supporting device. All images were analyzed using an automatic assessment method following the ACR MRI accreditation guidance. Image quality was compared between acquisitions grouped according to the angle of tilt of the coil supporting device: group A (Flat mode), group B (10˚), and group C (18˚). All measured image qualities were within the ACR recommended criteria, regardless of the angle of tilt of the flex tilt coil supporting device. However, statistically significant differences between the three groups were found for slice thickness, position accuracy, image intensity uniformity, and SNR (*P* < 0.05, ANOVA). The flex tilt coil supporting device can provide sufficient image quality, passing the criteria of the ACR MRI guideline, despite differences in slice thickness, slice position accuracy, image intensity uniformity, and SNR according to the angle of tilt.

## 1 INTRODUCTION

Modern MRI scanners are generally equipped with multichannel transmit‐receive coils of the birdcage design.^1^, ^2^, ^3^, ^4^ With the development of parallel imaging techniques, these coils provide uniform radiofrequency fields and spatially uniform image quality with a reduced scanning time.^5^, ^6^, ^7^, ^8^ However, these modern coils generally have a fixed‐geometry coil volume that is difficult to use with patients with kyphosis of the spine, who cannot lie completely flat during the MRI examination. For patients with such conditions, the conventional coil design causes patient discomfort and increases the rate of MRI examination failures. To address these problems, a nonmetallic flex tilt coil supporting device can provide an alternative geometry for the birdcage coil, allowing easier positioning and scanning of such patients. However, a coil supporting device tilts the coil off the isocenter in the anteroposterior direction, and the isocenter is one of the most important factors affecting image quality because the magnetic field degrades and gradient field nonlinearities increase with distance from the isocenter.^9^, ^10^, ^11^ Theoretically, imaging at or near the isocenter is desirable to produce high quality images, but it is not always possible during an MRI examination.

Up to the time of writing, there was no literature evaluating changes in image quality according to the angle of tilt of the coil supporting device. Therefore, the purpose of this study was to assess the effects on image quality of different angles of tilt created by a flex tilt coil supporting device during the MRI examination.

## 2 MATERIALS AND METHODS

### 2.A Phantom and study design

**Fig. 1:**
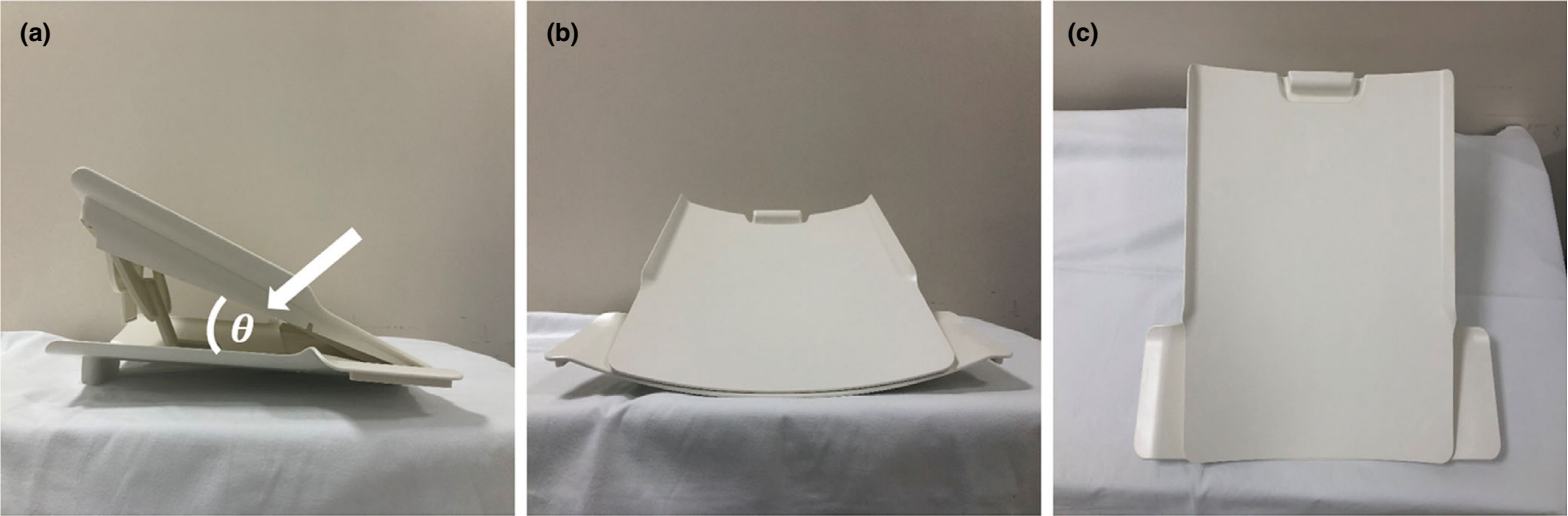
Images of the flex tilt coil supporting device and the angle of tilt. Theta (θ, white arrowhead) indicates the angle of tilt of the flex tilt coil. (a) lateral view of the supporting device, (b) anterior view, and (c) superior view.

An American College of Radiology (ACR; JM, Specialty Parts, San Diego, CA, USA) MRI phantom was used for the image analysis and measurements made in this study. This cylindrical phantom measures 148 mm in length and 190 mm in diameter, and contains a solution of nickel chloride and sodium (10 mM NiCl_2_ and 75 mM NaCl). The phantom was carefully aligned and positioned with the positioning indicator light aligned with its nose and chin landmarks. To align the center of the phantom with the isocenter of the scanner, the phantom was horizontally clamped by placing the cushion pads under either end of the head coil. The flex tilt coil was adjusted in three steps with angle of tilt (flat, 10°, and 18°) using a coil supporting device (Philips Healthcare, Fig. 1). Each angle of the flex tilt coil was measured with a protractor. The imaging data were divided into three groups according to the angle of tilt of the coil supporting device: group A (flat), group B (tilt 10°), and group C (tilt 18°).

### 2.B MR equipment and scan parameters

All images were acquired on a clinical 3.0‐T MRI scanner (Ingenia CX; Philips Healthcare, the Netherlands) with an 80 mT/m maximum gradient strength and a 200 T/m/s slew rate. Fifteen‐channel head coils (Philips Healthcare) were used for image acquisition. Two axial spin echo sequences were used to acquire T1‐weighted imaging (T1WI) and T2‐weighted imaging (T2WI), according to the standard sequence protocols prescribed by the ACR guideline.^12^ The uniformity correction mode CLEAR (scan option to improve the image intensity uniformity under Philips Healthcare) and adaptive radio‐frequency (RF) shimming were used for both T1WI and T2WI at a fixed and adequate bandwidth to reduce degradation of image quality and variation in RF nonuniformity. Both spin echo sequences were acquired in the axial plane based on the phantom frame of reference with the following parameters: field of view: 250 × 250 mm; voxel size: 1 × 1 mm; acquisition matrix: 256 × 256; number of excitations: 1; slice thickness: 5 mm; slice gap: 5 mm; number of slices: 11; receiver bandwidth: 218 Hz/pixel. Further description of the parameters is given in Table 1.

**Table 1:** Summary of the detailed image acquisition parameters in all groups.

	FOV (cm)	TR (ms)	TE (ms)	Matrix	Slice thickness/gap (mm)	NEX	BW (Hz)	Scan Time (min:s)
Localizer	25	200	20	256 × 256	20/2	1	218	0:53
T1	25	500	20	256 × 256	5/5	1	218	2:10
T2	25	2000	20/80	256 × 256	5/5	1	218	8:34

FOV, field of view; TR, repetition time; TE, echo time; NEX, number of excitations; BW, bandwidth.

### 2.C Image analysis

To analyze the quality of the acquired images, an ACR MRI quality control test consisting of eight quantitative tests was performed on seven sets of scans obtained under the same setup for each tilt angle using an open‐source Matlab code (R2016b; Mathworks, Natick, MA, USA) available from http://jidisun.wix.com/osaqa‐project/.^13^ The signal to noise ratio (SNR) measurement was performed using the subtraction method according to the following equation (an image subtraction was performed to produce a noise only image)^14^, ^15^:

(1) $$\mathrm{SNR}=\;\frac{S}{\sigma /\sqrt{2}}$$

where S is the mean signal value of the two images and σ is the standard deviation of the subtracted images. S and σ values were derived from the region of interest (ROI) encompassing 75% in the two images and the subtracted images. The SNR analysis was performed using Image J (Bethesda, MD, USA; http:// rsbweb.nih.gov/ij/).

Eight image parameters were evaluated: geometric accuracy, slice thickness accuracy, slice position accuracy, percentage intensity uniformity (PIU), percentage signal ghosting, SNR, low‐contrast object detectability, and high‐contrast spatial resolution.

### 2.D Statistical analysis

The Kolmogorov–Smirnov test was used to confirm that the eight measured parameters followed normal distributions. On the basis of the results of the Kolmogorov–Smirnov test, all seven parameters among the three groups were compared using analysis of variance (ANOVA). When statistically significant differences were demonstrated, post‐hoc tests were performed using the Tukey–Kramer method. Statistical analyses were performed using IBM SPSS Statistics for Windows/Macintosh, v. 21.0 (IBM Corp., Armonk, NY, USA). For all statistical analyses, a two‐sided level of *P* < 0.05 was considered statistically significant.

## 3 RESULTS

**Fig. 2:**
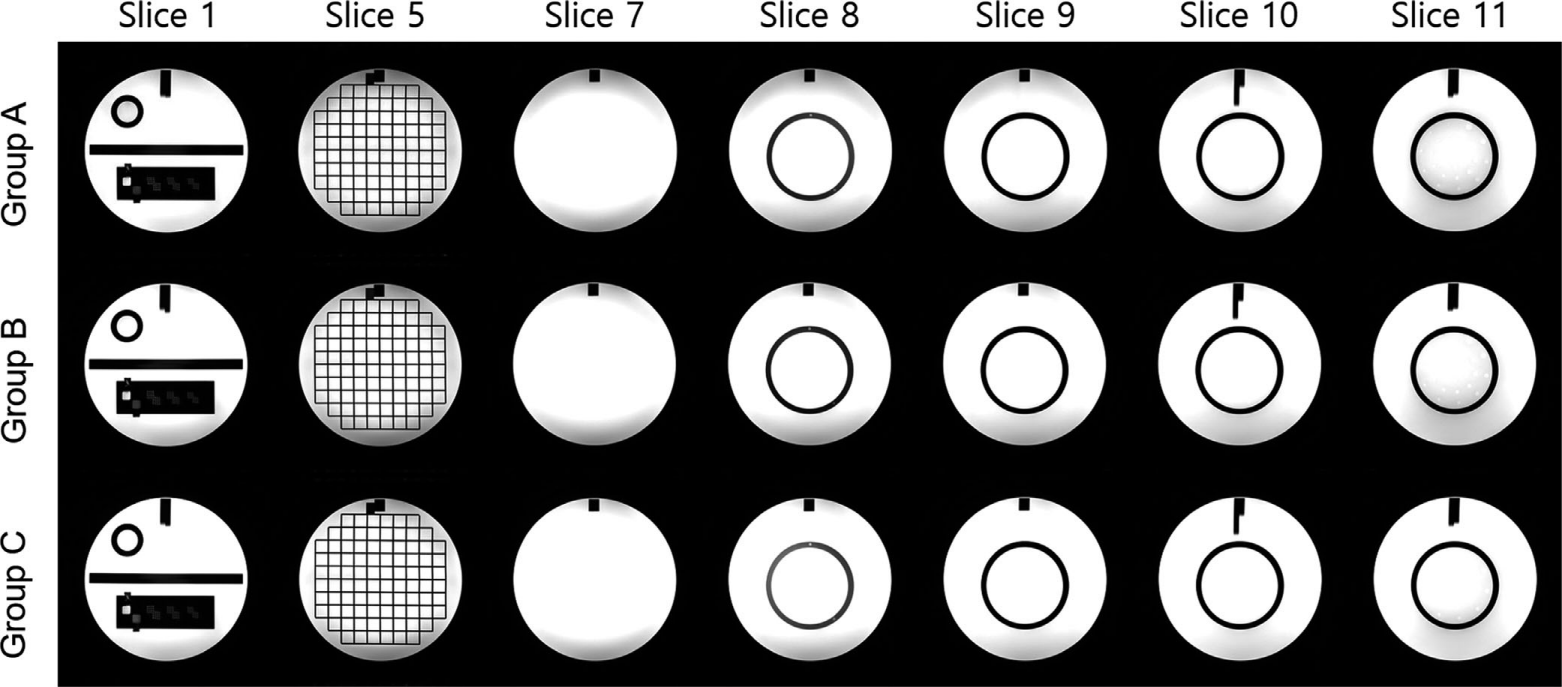
Representative images acquired with the flex tilt coil, displayed using their default contrast level and window. Group A, flat; Group B, tilt 10°; Group C, tilt 18°.

The measurements of the eight image parameters are presented in Table 2. Representative images acquired from all three groups are shown in Fig. 2. For geometric accuracy, all measured values were within the ACR criterion (±2 mm) for the true values. There were no statistically significant differences between groups A, B, and C in any direction (*P* > 0.05).

**Table 2:** Results of all eight image quality categories as a function of the angle of tilt.

Test	Slice and index	Group A (Flat mode)	Group B (Tilt 10°)	Group C (Tilt 18°)	*P*‐value
Geometric accuracy (mm)	T1 localizer	147.6 ± 0.2	147.8 ± 0.1	147.8 ± 0.1	0.225
	T1 slice 1 (AP)	189.2 ± 0.2	189.2 ± 0.2	189.1 ± 0.1	0.679
	T1 slice 1 (LR)	189.6 ± 0.3	189.4 ± 0.2	189.3 ± 0.1	0.339
	T1 slice 5 (AP)	188.8 ± 0.2	189.4 ± 0.4	189.2 ± 0.4	0.088
	T1 slice 5 (LR)	189.2 ± 0.3	189.1 ± 0.3	189.1 ± 0.4	0.931
	T1 slice 5 (UL to LR)	189.2 ± 0.2	189.3 ± 0.3	189.1 ± 0.2	0.845
	T1 slice 5 (UR to LL)	189.4 ± 0.2	189.2 ± 0.4	188.9 ± 0.4	0.101
High‐contrast spatial resolution	T1 slice 1 (UL)	0.9	0.9	0.9	NA
	T1 slice 1 (LR)	0.9	0.96 ± 0.05	0.9	0.109
	T2 slice 1 (UL)	0.9	0.9	0.9	NA
	T2 slice 1 (LR)	0.9	0.93 ± 0.04	0.9	0.483
Slice thickness accuracy (mm)	T1 slice 1	4.88 ± 0.01	4.96 ± 0.02	4.84 ± 0.02	< 0.05*^‡^
	T2 slice 1	5.02 ± 0.07	5.01 ± 0.09	4.99 ± 0.02	0.736
Slice position accuracy (mm)	T1 slice 1	1.85 ± 0.08	2.88 ± 0.03	1.94 ± 0.13	< 0.05*^‡^
	T1 slice 11	−2.67 ± 0.03	−2.93 ± 0.02	−2.99 ± 0.15	< 0.05*^†^
	T2 slice 1	1.01 ± 0.09	2.88 ± 0.06	1.99 ± 0.08	< 0.05*^†‡^
	T2 slice 11	−2.91 ± 0.09	−1.97 ± 0.05	−2.85 ± 0.08	< 0.05*^‡^
Image intensity uniformity (%)	T1 slice 7	84.72 ± 1.12	90.71 ± 0.65	89.99 ± 0.36	< 0.05*^†^
	T2 slice 7	85.09 ± 0.51	90.44 ± 0.27	89.97 ± 0.32	< 0.05*^†^
Signal to noise ratio	T1 slice 7	941.49 ± 17.52	965.13 ± 12.28	953.11 ± 14.63	0.273
	T2 slice 7	642.97 ± 9.16	690.17 ± 8.56	671.58 ± 8.24	< 0.05*^†^
Percent‐signal ghosting	T1 slice 7	0.0023 ± 0.0002	0.0024 ± 0.0001	0.0021 ± 0.0004	0.508
Low‐contrast object detectability	T1 slices 8–11 (#spokes)	39.6 ± 0.4	38.6 ± 0.4	38.6 ± 0.5	0.09
	T2 slices 8–11 (#spokes)	38.3 ± 0.4	38.3 ± 0.4	37.6 ± 0.4	0.392

Statistically significant differences are demonstrated using the Tukey–Kramer post‐hoc method. *indicates *P*‐values between flat and 10° tilt, †between flat and 18° tilt, and ‡between 10° and 18° tilt. NA, not applicable.

**Fig. 3:**
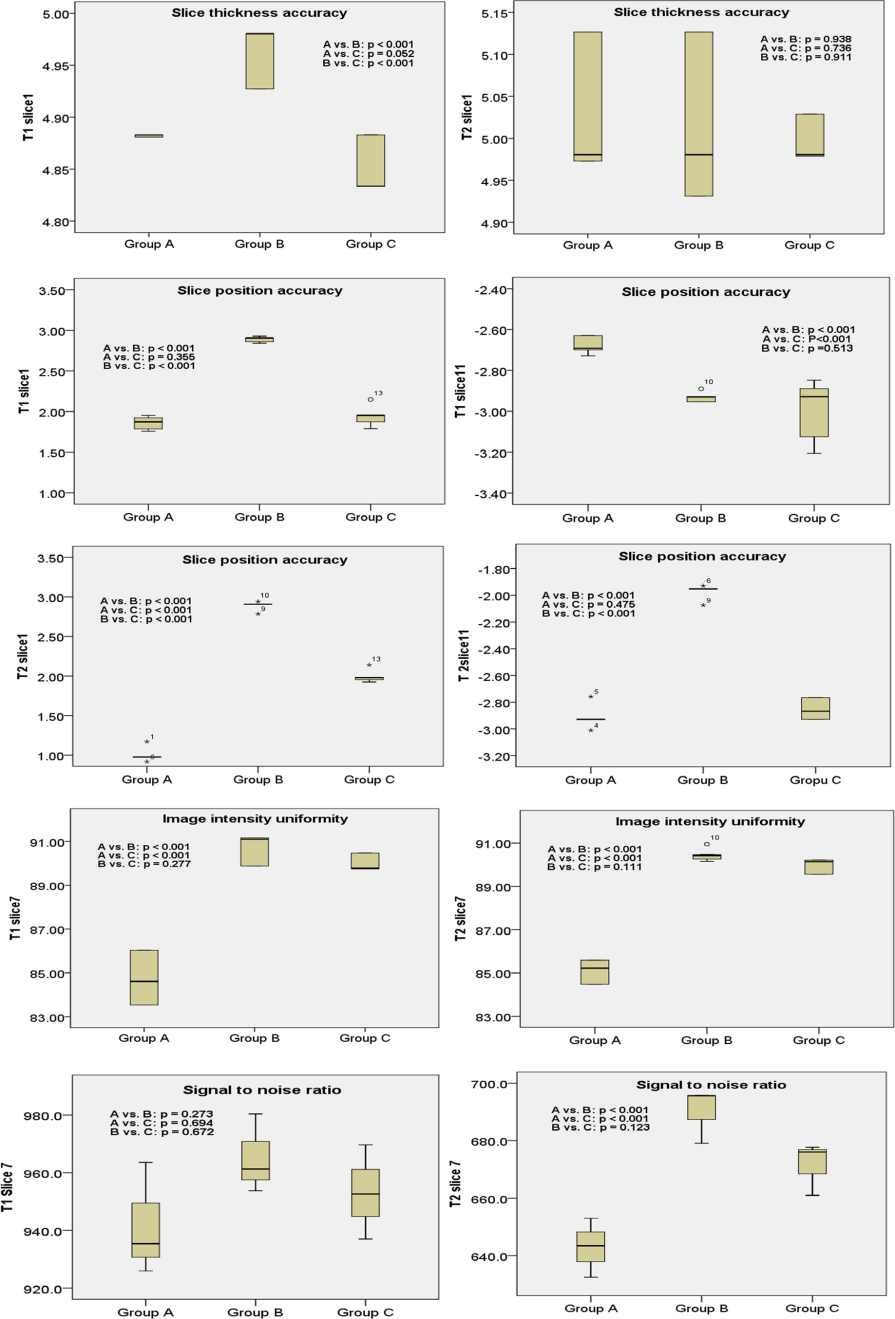
Box plots showing statistically differences in image quality categories between groups A, B, and C.

The spatial resolution of both slice 1 images of T1WI and T2WI in both directions passed the ACR criterion of 1.0 mm in all three groups. No statistically significant differences in either slice 1 of T1WI or T2WI in either direction were found between the groups (*P* > 0.05). For slice thickness accuracy, all measured values in all three groups were within the ACR criterion of 5.0 ± 0.7 mm. There were no significant differences in slice 1 of T2WI between groups A, B, and C (*P* > 0.05). In slice 1 of T1WI, no statistically significant differences were found between groups A and C (*P* = 0.052) (Fig. 3).

For slice position accuracy, all measured values were within the ACR criterion of 5 mm or less of the absolute value. There was no significant difference between groups A and C for either slice 1 of T1WI or slice 11 of T2WI (*P* = 0.355 and *P* = 0.475, respectively). In addition, there was no statistically significant difference between groups B and C in slice 11 of T1WI (*P* = 0.513). However, there were significant differences between groups A, B, and C for slice 1 of T2WI (*P* < 0.05) (Fig. 3).

For image intensity uniformity, there were no statistically significant differences in either slice 7 of T1WI or slice 7 of T2WI between groups B and C (*P* = 0.277 and *P* = 0.111, respectively) (Fig. 3). In addition, all measured image intensity uniformities were greater than the ACR criterion of 82% for 3.0 T. For SNR, there were no statistically significant differences in slice 7 of T1WI between groups A, B, and C (*P* > 0.05) or in slice 7 of T2WI between groups B and C (*P* = 0.123). For percent‐signal ghosting, there were no significant differences between groups A, B, and C (*P* > 0.05). All measured ghosting ratios were less than the ACR criterion of 0.025.

For low‐contrast object detectability, the total number of measured spokes in all three groups passed the ACR criterion of greater than 37 spokes for 3.0 T. There were no statistically significant differences between groups A, B, and C for either T1WI or T2WI (*P* > 0.05). However, the total number of measured spokes tended to decrease as the angle of tilt increased.

## 4 DISCUSSION

With the increasing use of well‐designed vender‐specific supporting devices for precise examinations, a qualitative and quantitative analysis of the effects of these devices on image quality is important. However, the effects are often underestimated, and their effectiveness is rarely demonstrated in clinical practice. Our results showed that seven routine ACR quality assurance (QA) tests showed acceptable image quality within the ACR recommended criteria for all images, regardless of the angle of tilt of the flex tilt coil supporting device. This means that the flex tilt coil supporting device can help patients with kyphosis of the spine more comfortable undergoing examination while maintaining image quality.

When a phantom is used for a quantitative and qualitative image quality analysis, it is important to indicate definite and objective criteria. In addition, the QA process should be easy to perform and as simple and convenient as possible. Previous studies used a standard set of image QA procedures using numerous phantoms.^16^, ^17^, ^18^ However, manual assessment methods appear to be complicated and inefficient for assessing image quality, tending to be highly dependent on the observer or monitor, and also time consuming. There may also be the problem that contrast evaluation, which is considered to be a crucial image quality category, is not performed. On the other hand, some studies have demonstrated automatic assessment methods to reduce the QA processing time while improving the consistency and objectivity of measured values.^13^, ^19^, ^20^ Thus, we also used automatic image quality measurements available through an open‐source code, measurements that were relatively easy to perform in the current study.

Our results showed that most values of the categories evaluated were similar, regardless of the angle of tilt of the flex tilt coil. However, some comparisons revealed statistically significant differences in slice thickness, slice position accuracy, and image intensity uniformity. These can be explained by magnetic field inhomogeneity and gradient field nonlinearity caused by moving away from the isocenter of the magnet bore, with these causing image distortion and degradation. A few studies showed that magnetic field inhomogeneity and gradient field nonlinearity increase toward the periphery away from the magnet isocenter, thus leading to nonuniform intensity.^9^, ^11^, ^21^ As well, differences in the measured values resulting from changes in the angle of tilt coil and phantom positioning may influence the RF shimming coefficients required to achieve the most uniform B_1_ field inside phantom. Some studies demonstrated that adaptive RF shimming has the potential to improve RF homogeneity.^22^, ^23^ Thus, the use of adaptive RF shimming is important to mitigate the variation of RF nonuniformity. Despite the application of this method, some results showed statistically significant differences. In addition, differences in slice position accuracy might be explained by the fact that it was difficult to accurately and consistently position the ACR phantom across the different angles of tilt.

Some researchers reported that it is essential to use intensity correction to improve the homogeneity and uniformity of images when using a multichannel coil.^13^, ^24^ This is because multichannel phased‐array head coils have smaller coil elements that produce a less uniform image in comparison with quadrature coils. In this study, we used the vendor’s intensity correction mode to improve image uniformity, and the results stated above reflect the use of this intensity correction. We consider it worth noting that our study describes the bandwidth, which has an effect on image quality, unlike other publications evaluating image quality with the ACR phantom. Only one previous study reported bandwidth, and its value (150 Hz) was similar to ours (218 Hz).^24^ It is widely known that a narrower bandwidth theoretically results in higher image distortion. However, a larger receiver bandwidth causes degradation of the signal‐to‐noise ratio (SNR) because of the inclusion of more noise. Thus, the bandwidth was considered adequate to reduce degradation of image quality in the current study.

There are some limitations to our study. First, the results of this study were only from investigations using the ACR phantom. This specific phantom is used for quality control and system performance testing, and obviously does not represent the diversity of patients who cannot comfortably lie flat and those with kyphosis of the spine. As the eight measurements are conducted on different image slices that are at different distances from the isocenter of the scanner, different measurements are subjected to different amount of influence from tiling. In addition, the imaged slices covered only a range of 11cm which was considerable shorter than coverage of a typical head coil. As a results, this study provides only limited value to the understanding of influence of tilting on the image quality. Therefore, a further study is needed to assess image quality with a diverse range of body shapes to demonstrate the effectiveness and objectiveness of the flex tilt coil supporting device in practice. Second, we only used T1WI and T2WI without applying other sequences. Because the ACR protocol can clearly demonstrate image quality based on well‐organized evaluation categories according to the ACR MR phantom guideline. Nevertheless, this is the first research to focus on comparing image quality according to the angle of tilt of the coil supporting device.

## 5 CONCLUSIONS

The flex tilt coil supporting device can provide sufficient image quality passing the criteria of the ACR MR phantom guideline, despite differences in slice thickness, slice position accuracy, image intensity uniformity, and SNR according to the angle of tilt.

## CONFLICT OF INTEREST

No conflict of interest.

## Author Contribution

Sung Min Kim guarantors of integrity of entire study. Ji Sung Jang and Ho beom Lee were involved in study concepts and data acquisition. Ho beom Lee and Ki Baek Lee were involved in data analysis and statistical analysis. All authors were involved in literature research.
